# Editorial for the Special Issue on Quantum Dots Frontiers

**DOI:** 10.3390/mi14051026

**Published:** 2023-05-10

**Authors:** Wei Chen, Junjie Hao

**Affiliations:** 1 Shenzhen Key Laboratory of Ultraintense Laser and Advanced Material Technology, Center for Advanced Material Diagnostic Technology, College of Engineering Physics, Shenzhen Technology University, Shenzhen 518118, China; 2 College of Integrated Circuits and Optoelectronic Chips, Shenzhen Technology University, Shenzhen 518118, China

Thanks to state-of-the-art chemical and device engineering in past decades, we have witnessed more and more novel applications based on semiconductor nanocrystals: quantum dots (QDs). Besides the applications on bio-labeling [1], QDs have already exhibited great commercial value in lighting applications due to their unique properties compared to traditional technologies. For instance, the emission of QDs is bright, tunable in a visible range, and narrow in bandwidth, enabling high-quality and low-cost lighting including high-color-rendering lights and wide-color-gamut display backlights (known as photoluminescence (PL)-type QD applications) [2,3,4]. On this basis, with ligand engineering and refined encapsulation, PL-type QD applications have successfully inspired lighting and display commercialization. Moreover, based on efforts of device engineering, electroluminescence (EL)-type QDs applications, known as QD-light-emitting diode (or QLED), are also very close to commercialization, particularly for green and red QLED. The main challenge remaining is achieving efficient and stable QLED with blue emission. Notably, the QDs for lighting and display applications are mainly referred to CdSe QDs, InP QDs (mostly merged as core-shell structured QDs, and the alloyed QDs) [3,5,6,7], and perovskite nanocrystals [8,9,10].

Another branch of QD applications is based on the unique properties of QDs: the solution processibility and the broad wavelength spectral response. In this branch, the most representative QDs are lead chalcogenides QDs, PbS, and PbSe, which bring new opportunities for fabricating low-cost and high-resolution short-wave infrared (SWIR) imagers. The processibility of these QDs enables direct integration with the present CMOS technique and thus significantly reduces the price of SWIR imagers [11,12,13]. The key component of the SWIR imager is known as a diode-type photodetector (PD) array in which a single pixel exhibits a similar device architecture to a photovoltaic device [14,15]. In such a charge extraction application, the QDs are atomically surface passivated in the film, working as both a light absorber for charge generation and a charge carrier transport medium. Therefore, the surface passivation and the inter-dot distance-dominated stacking configuration of the QDs are both important to stable and efficient solid films in such applications [16].

Overall, QDs, as novel materials with a large surface-to-volume ratio, are sensitive to ambient conditions. Thus, to address different application requests, the type of QDs and the treatments can be fundamentally different. In this Special Issue on “Quantum Dot Frontiers”, we included nine papers on different QDs with different application backgrounds. Specifically, for QDs in material science, Hu et al. investigated the properties of doped perovskite QDs with mixed-A-cations [10]. Moreover, Yoo et al. improved the efficiency of cadmium-free QDs via advanced surface passivation [6]. Halim et al. investigated the electron–phonon coupling mechanism of PbS QDs and PbS/MnTe QDs by tracking their temperature-dependent PL spectra [17]. For the bio-labeling application, Le et al. employed core-shell structured QDs in detecting doxycycline in nature water and in food [1]. For PL-type QD applications, Xiao et al. presented a method to optimize the light quality of white-light LED, in which the QD-based white-light LED is also involved [2]. Zhao et al. reported high-resolution QD arrays in micro-LED applications [3]. Towards EL-type QD applications, Ye et al. reported an efficient QLED device with the perovskite QD active layer deposited via an ink-jet method [8]. To improve the QLED performance, Wang et al. introduced a novel organic hole transport layer material in the QLED fabrication process [9]. A flexible QLED device exhibiting an exemplary performance compared with the rigid device was demonstrated by Kim et al. by employing novel device components [5]. We believe that based on further developments in chemistry engineering and device engineering, the era of QD applications is approaching.

## References

[1] Le T., Xu R., Yang L., Xie Y. (2022). Development of a Highly Specific Fluoroimmunoassay for the Detection of Doxycycline Residues in Water Environmental and Animal Tissue Samples. Micromachines.

[2] Xiao H., Li Y., Li B., Wang G. (2022). An Investigation on CCT and Ra Optimization for Trichromatic White LEDs Using a Dual-Weight-Coefficient-Based Algorithm. Micromachines.

[3] Zhao B., Wang Q., Li D., Yang H., Bai X., Li S., Liu P., Sun X. (2022). Red and Green Quantum Dot Color Filter for Full-Color Micro-LED Arrays. Micromachines.

[4] Chen W., Wang K., Hao J., Wu D., Qin J., Dong D., Deng J., Li Y., Chen Y., Cao W. (2016). High Efficiency and Color Rendering Quantum Dots White Light Emitting Diodes Optimized by Luminescent Microspheres Incorporating. Nanophotonics.

[5] Kim M., Kim D., Kwon O., Lee H. (2022). Flexible CdSe/ZnS Quantum-Dot Light-Emitting Diodes with Higher Efficiency Than Rigid Devices. Micromachines.

[6] Yoo D., Bak E., Ju H.M., Shin Y.M., Choi M.J. (2022). Zinc Carboxylate Surface Passivation for Enhanced Optical Properties of In(Zn)P Colloidal Quantum Dots. Micromachines.

[7] Zhang W., Duan X., Tan Y., Liu H., Hao J., Chen W., Liu P., Wang K., Yang X., Xu W. (2022). Organic-Phase Synthesis of Blue Emission Copper Nanoparticles for Light-Emitting Diodes. ACS Appl. Nano Mater..

[8] Ye T., Jia S., Wang Z., Cai R., Yang H., Zhao F., Tan Y., Sun X., Wu D., Wang K. (2022). Fabrication of Highly Efficient Perovskite Nanocrystal Light-Emitting Diodes Via Inkjet Printing. Micromachines.

[9] Wang W., Li Y., Duan Y., Qiu M., An H., Peng Z. (2022). Performance Enhancement of Perovskite Quantum Dot Light-Emitting Diodes Via Management of Hole Injection. Micromachines.

[10] Hu L., Zhao W., Duan W., Chen G., Fan B., Zhang X. (2022). Temperature-Dependent Optical Properties of Perovskite Quantum Dots with Mixed-a-Cations. Micromachines.

[11] Liu J., Liu P., Chen D., Shi T., Qu X., Chen L., Wu T., Ke J., Xiong K., Li M. (2022). A near-Infrared Colloidal Quantum Dot Imager with Monolithically Integrated Readout Circuitry. Nat. Electron..

[12] Pejovic V., Lee J., Georgitzikis E., Li Y., Kim J.H., Lieberman I., Malinowski P.E., Heremans P., Cheyns D. (2021). Thin-Film Photodetector Optimization for High-Performance Short-Wavelength Infrared Imaging. IEEE Electron Device Lett..

[13] Pejovic V., Georgitzikis E., Lee J., Lieberman I., Cheyns D., Heremans P., Malinowski P.E. (2022). Infrared Colloidal Quantum Dot Image Sensors. IEEE Trans. Electron Devices.

[14] Chen W., Guo R., Tang H., Wienhold K.S., Li N., Jiang Z., Tang J., Jiang X., Kreuzer L.P., Liu H. (2021). Operando Structure Degradation Study of PbS Quantum Dot Solar Cells. Energ. Environ. Sci..

[15] Vafaie M., Fan J.Z., Morteza Najarian A., Ouellette O., Sagar L.K., Bertens K., Sun B., García de Arquer F.P., Sargent E.H. (2021). Colloidal Quantum Dot Photodetectors with 10-ns Response Time and 80% Quantum Efficiency at 1550 nm. Matter.

[16] Xia Y., Chen W., Zhang P., Liu S., Wang K., Yang X., Tang H., Lian L., He J., Liu X. (2020). Facet Control for Trap-State Suppression in Colloidal Quantum Dot Solids. Adv. Funct. Mater..

[17] Halim N.D., Zaini M.S., Talib Z.A., Liew J.Y.C., Kamarudin M.A. (2022). Study of the Electron-Phonon Coupling in PbS/MnTe Quantum Dots Based on Temperature-Dependent Photoluminescence. Micromachines.

